# Genetic Architecture of Salt Tolerance in Cowpea (*Vigna unguiculata* (L.) Walp.) at Seedling Stage Using a Whole Genome Resequencing Approach

**DOI:** 10.3390/ijms242015281

**Published:** 2023-10-18

**Authors:** Waltram Ravelombola, Lindgi Dong, Thomas Casey Barickman, Haizheng Xiong, Aurora Manley, John Cason, Hanh Pham, Bazgha Zia, Beiquan Mou, Ainong Shi

**Affiliations:** 1 Texas A&M AgriLife Research, 11708 Highway 70 South, Vernon, TX 76384, USA; aurora.manley@ag.tamu.edu; 2 Department of Soil and Crop Sciences, Texas A&M University, 370 Olsen Blvd., College Station, TX 77843, USA; 3 Institute of Cash Crops, Hebei Academy of Agriculture and Forestry Sciences, Shijiazhuang 050051, China; 4 North Mississippi Research and Extension Center, Department of Plant and Soil Sciences, Mississippi State University, Verona, MS 38879, USA; 5 Department of Horticulture, University of Arkansas, Fayetteville, AR 72701, USA; 6 Texas A&M AgriLife Research, 1129 North US Highway 281, Stephenville, TX 76401, USA; john.cason@ag.tamu.edu; 7 Texas A&M AgriLife Research, 1102 East Drew Street, Lubbock, TX 79403, USA; 8 United States Vegetable Lab (USVL), 2700 Savannah Hwy, Charleston, SC 29414, USA; 9 Agricultural Research Service (USDA ARS), 1636 E. Alisal St., Salinas, CA 93905, USA; beiquan.mou@usda.gov

**Keywords:** cowpea, seedling, salt, GWAS, whole genome

## Abstract

Cowpea (*Vigna unguiculata* (L.) Walp.) is a diploid legume crop used for human consumption, feed for livestock, and cover crops. Earlier reports have shown that salinity has been a growing threat to cowpea cultivation. The objectives of this study were to conduct a genome-wide association study (GWAS) to identify SNP markers and to investigate candidate genes for salt tolerance in cowpea. A total of 331 cowpea genotypes were evaluated for salt tolerance by supplying a solution of 200 mM NaCl in our previous work. The cowpea panel was genotyped using a whole genome resequencing approach, generating 14,465,516 SNPs. Moreover, 5,884,299 SNPs were used after SNP filtering. GWAS was conducted on a total of 296 cowpea genotypes that have high-quality SNPs. BLINK was used for conducting GWAS. Results showed (1) a strong GWAS peak on an 890-bk region of chromosome 2 for leaf SPAD chlorophyll under salt stress in cowpea and harboring a significant cluster of nicotinamide adenine dinucleotide (NAD) dependent epimerase/dehydratase genes such as *Vigun02g128900.1*, *Vigun02g129000.1*, *Vigun02g129100.1*, *Vigun02g129200.1*, and *Vigun02g129500.1*; (2) two GWAS peaks associated with relative tolerance index for chlorophyll were identified on chromosomes 1 and 2. The peak on chromosome 1 was defined by a cluster of 10 significant SNPs mapped on a 5 kb region and was located in the vicinity of *Vigun01g086000.1*, encoding for a GATA transcription factor. The GWAS peak on chromosome 2 was defined by a cluster of 53 significant SNPs and mapped on a 68 bk region of chromosome 2, and (3) the highest GWAS peak was identified on chromosome 3, and this locus was associated with leaf score injury. This peak was within the structure of a potassium channel gene (*Vigun03g144700.1*). To the best of our knowledge, this is one the earliest reports on the salt tolerance study of cowpea using whole genome resequencing data.

## 1. Introduction

Salt stress is a growing threat affecting cowpea production worldwide [1,2]. Previous studies have shown salt stress resulting in significant crop yield losses [3,4,5]. Soil salinity affects over 830 million hectares of croplands worldwide [6,7,8]. In the U.S., soil salinity has been reported on over 19.6 million hectares of croplands. The cost associated with soil salinity is estimated to be 12 billion USD annually [9,10,11]. In addition, irrigation water from rivers in semi-arid croplands can rapidly increase soil degradation through a continuous accumulation of salt [12].

Effects of salinity have been shown to be increasingly more insidious in semi-arid areas where cowpea cultivation is prevalent [13]. For example, cowpea grown in the Coachella Valley of California has been negatively affected by salinity [14]. Salinity can lead to significant plant phytological and morphological damage to cowpea plants [15]. At the seedling stage, salinity reduces the photosynthetic activity of cowpea plants, where excess of Na^+^ in plant tissue can lead to plant death [16,17]. The negative impacts of salinity on crops are more significant in semi-arid and arid regions where cowpea cultivation is prevalent, which could refrain growers from expanding areas for cowpea production [18]. Salt stress reduced cowpea plant vigor and yield-reducing for cowpea [19]. Salt stress can also cause a significant decrease in yield for cowpea grown on calcareous soils [20]. The seedling stage has been found to be the most vulnerable stage to salt stress in cowpea [8]. Therefore, evaluating salt tolerance at the seedling stage and mapping QTL alleles for salt tolerance at the seedling stage are needed.

DNA sequencing by the next-generation sequencing technologies (van Dijk et al., 2014), using improved double digest Restriction Site Associated DNA sequencing (ddRADseq) [21] and Genotyping by Sequencing (GBS) [22] is used for single nucleotide polymorphism (SNP) discovery and genotyping. GBS and ddRADseq are affordable ways to develop SNPs and SNP genotyping, establish genetic maps, and map QTLs [22]. However, both GBS and ddRADseq have disadvantages, such as untargeted and large amounts of missing data across given taxonomic samples due to their reduced genome sequencing. With the decrease in DNA sequencing cost, whole-genome sequencing (WGS) and whole-genome resequencing (WGR) provide opportunities to develop a large number of SNPs [23]. WGS can generate whole-genome assemblies (genome sequences) for any plant with and without genome information through de novo assembly [22]. WGR provides a high-resolution, base-by-base view of the genome. In addition, the WGR is widely used to detect single nucleotide variants (SNVs), including SNPs, insertions and deletions (InDels), structural variants (S.V.s), and copy number variation (CNV). It also allows the examination of SNVs, InDels, S.V.s, and CNVs in the genome’s coding and non-coding regions with reliable sequence coverage and coverage uniformity at whole-genome level (http://www.illumina.com/techniques/sequencing/dna-sequencing/whole-genome-sequencing.html (accessed on 3 March 2020)). Therefore, we will use the WGR technology for genotyping and SNP discovery for cowpea salt tolerance in this study.

We have done several experiments for salt tolerance in cowpea at the germination and seedling stages [24]. In one of the previous experiments, 155 cowpea genotypes as an association panel were phenotyped for foliar injury, plant height, and fresh and dry shoot weight under 0 mM and 200 mM NaCl (without salt stress and salt treatment) conditions and genotyped using 1049 SNPs postulated from genotype by sequencing (GBS). Association study in this panel showed three SNP markers, Scaffold87490_622, Scaffold87490_630, and C35017374_128, associated with salt tolerance at the germination stage, and seven SNP markers, Scaffold93827_270, Scaffold68489_600, Scaffold87490_633, Scaffold87490_640, Scaffold82042_3387, C35069468_1916, and Scaffold93942_1089, associated with salt tolerance at seedling stage [24]. 

In this study, we conduct GWAS with 331 cowpea genotypes as an association panel for salt tolerance at the early seedling stage to identify SNP markers associated with salt tolerance in cowpea using WGR technology. The phenotypic data of salt tolerance in the 331 cowpea genotypes have been published [25]. Here, we will report SNP markers and candidate genes for salt tolerance in cowpea.

## 2. Results

### 2.1. Leaf SPAD Chlorophyll under Salt Stress

**Figure 1:**
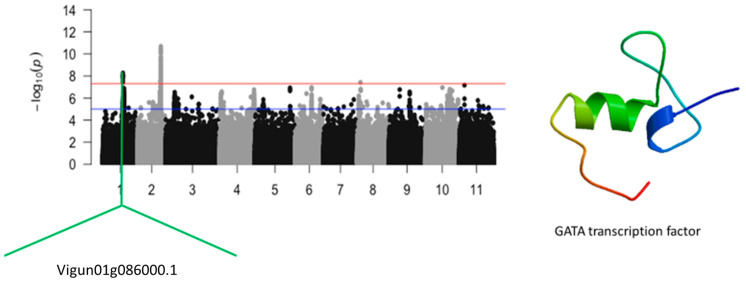
Manhattan plot for leaf SPAD chlorophyll under salt stress in cowpea. The solid black and grey dots represent the SNPs. The *x*-axis is the chromosome number, and the *y*-axis is the LOD or −log10 of the *p*-value. The horizontal red and blue bars are two different LOD thresholds. Below the Manhattan plot are gene IDs from phytozome v.13 (https://phytozome.jgi.doe.gov/pz/portal.html (accessed on 3 March 2020)) corresponding to the significant locus on chromosome 1. Codifying sequences of the gene IDs whose functions were related to drought stress were extracted and converted to amino acid sequences using BLASTX (https://blast.ncbi.nlm.nih.gov/Blast.cgi (accessed on 3 March 2020)). Tertiary structures of the proteins/polypeptides derived from BLASTX were predicted using SWISS-MODEL (https://swissmodel.expasy.org/ (accessed on 3 March 2020)) and presented on the left-hand side in the above figure.

**Figure 2:**
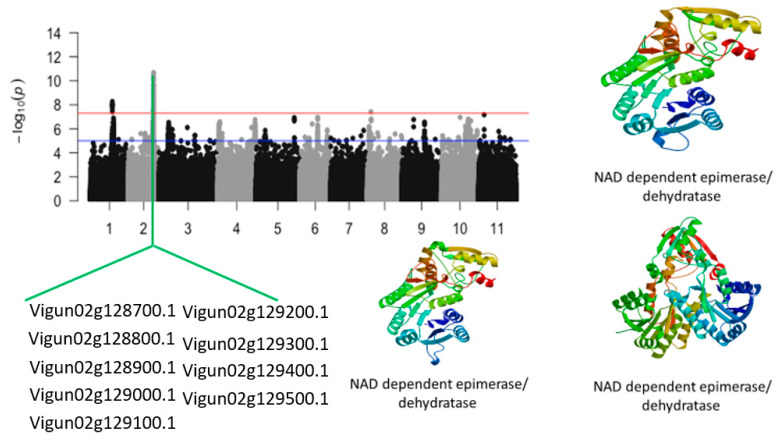
Manhattan plot for leaf SPAD chlorophyll under salt stress in cowpea. The solid black and grey dots represent the SNPs. The *x*-axis is the chromosome number, and the *y*-axis is the LOD or −log10 of the *p*-value. The horizontal red and blue bars are two different LOD thresholds. Below the Manhattan plot are gene IDs from phytozome v.13 (https://phytozome.jgi.doe.gov/pz/portal.html (accessed on 3 March 2020)) corresponding to the significant locus on chromosome 2. Codifying sequences of the gene IDs whose functions were related to drought stress were extracted and converted to amino acid sequences using BLASTX (https://blast.ncbi.nlm.nih.gov/Blast.cgi (accessed on 3 March 2020)). Tertiary structures of the proteins/polypeptides derived from BLASTX were predicted using SWISS-MODEL (https://swissmodel.expasy.org/ (accessed on 3 March 2020)) and presented on the left-hand side in the above figure.

Results indicated that a total of 65 SNPs were significantly associated with leaf SPAD chlorophyll under salt stress in cowpea (Figure 1 and Figure 2). These SNPs were located on chromosomes 1 and 2. Chromosome 1 harbored a total of 9 significant SNPs, whereas chromosome 2 had a total of 56 significant SNPs (Table S1). LOD (−log_10_(*p*-value)) values varied from 7.53 to 10.68. The first locus that was identified to be associated with leaf SPAD chlorophyll under salt stress was defined by a cluster of significant SNPs mapped on a 3 kb region of chromosome 1. The second locus that was found to be associated with leaf SPAD chlorophyll under salt stress was defined by a group of significant SNPs mapped on an 890 kb region of chromosome 2. The significant SNPs that were found on chromosome 1 were Vu01_24245081 (LOD = 7.57), Vu01_24246312 (LOD = 8.00), Vu01_24246319 (LOD = 8.00), Vu01_24246550 (LOD = 7.76), Vu01_24246587 (LOD = 7.94), Vu01_24246822 (LOD = 8.27), Vu01_24246905 (LOD = 8.08), Vu01_24246981 (LOD = 8.07), and Vu01_24248242 (LOD = 7.85) (Figure 1). The SNP that was closest to an annotated gene, *Vigun01g086000.1*, was Vu01_24245081. *Vigun01g086000.1* encodes for the GATA transcription factor whose predicted tertiary structure is shown in Figure 1. 

The second locus, defined by an 890 kb region of chromosome 2 harbored nine annotated genes. Table S1 shows that the SNPs with the highest LOD (−log_10_(*p*-value)) were Vu02_28054154 (LOD = 10.68), Vu02_28050297 (LOD = 10.45), Vu02_28050011 (LOD = 10.26), Vu02_28050187 (LOD = 10.22), Vu02_28105724 (LOD = 10.05), Vu02_28105725 (LOD = 10.05), Vu02_28094085 (LOD = 9.71), Vu02_28084764 (LOD = 9.63), Vu02_28068945 (LOD = 9.61), Vu02_28054571 (LOD = 9.56), Vu02_28044965 (LOD = 9.43), Vu02_28064123 (LOD = 9.37), Vu02_28069038 (LOD = 9.33), Vu02_28067838 (LOD = 9.31), Vu02_28090457 (LOD = 9.25), Vu02_28064103 (LOD = 9.01), Vu02_28090387 (LOD = 8.93), and Vu02_28052297 (LOD = 8.91). The SNPs that were in the vicinity or within the structure of candidate genes were Vu02_28035590 (LOD = 8.33), Vu02_28044965 (LOD = 9.43), Vu02_28050297 (LOD = 10.45), Vu02_28054154 (LOD = 10.68), Vu02_28064103 (LOD = 9.01), Vu02_28068945 (LOD = 9.61), Vu02_28084764 (LOD = 9.63), Vu02_28090457 (LOD = 9.25), and Vu02_28105724 (LOD = 10.05) (Table 1). These SNPs were within or close to *Vigun02g128700.1*, *Vigun02g128800.1*, *Vigun02g128900.1*, *Vigun02g129000.1*, *Vigun02g129100.1*, *Vigun02g129200.1*, *Vigun02g129300.1*, *Vigun02g129400.1*, and *Vigun02g129500.1*. The candidate genes consisted of a cluster of NAD-dependent epimerase/dehydratase whose predicted tertiary structure is shown in Figure 2.

### 2.2. Relative Tolerance Index for Chlorophyll Content

**Figure 3:**
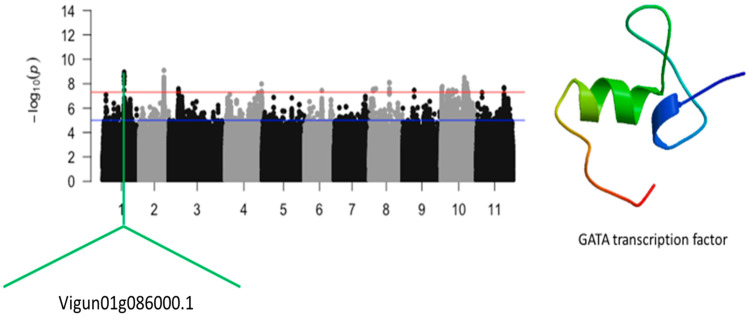
Manhattan plot relative tolerance index for leaf SPAD chlorophyll for salt stress in cowpea. The solid black and grey dots represent the SNPs. The *x*-axis is the chromosome number, and the *y*-axis is the LOD or −log10 of the *p*-value. The horizontal red and blue bars are two different LOD thresholds. Below the Manhattan plot are gene IDs from phytozome v.13 (https://phytozome.jgi.doe.gov/pz/portal.html (accessed on 3 March 2020)) corresponding to the significant locus on chromosome 1. Codifying sequences of the gene IDs whose functions were related to drought stress were extracted and converted to amino acid sequences using BLASTX (https://blast.ncbi.nlm.nih.gov/Blast (accessed on 3 March 2020)). Tertiary structures of the proteins/polypeptides derived from BLASTX were predicted using SWISS-MODEL (https://swissmodel.expasy.org/ (accessed on 3 March 2020)) and presented on the left-hand side in the above figure.

**Figure 4:**
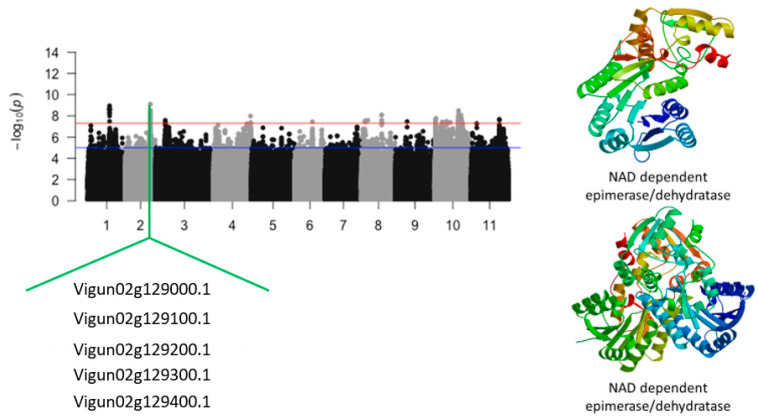
Manhattan plot relative tolerance index for leaf SPAD chlorophyll for salt stress in cowpea. The solid black and grey dots represent the SNPs. The *x*-axis is the chromosome number, and the *y*-axis is the LOD or −log10 of the *p*-value. The horizontal red and blue bars are two different LOD thresholds. Below the Manhattan plot are gene IDs from phytozome v.13 (https://phytozome.jgi.doe.gov/pz/portal.html (accessed on 3 March 2020)) corresponding to the significant locus on chromosome 2. Codifying sequences of the gene IDs whose functions were related to drought stress were extracted and converted to amino acid sequences using BLASTX (https://blast.ncbi.nlm.nih.gov/Blast.cgi (accessed on 3 March 2020)). Tertiary structures of the proteins/polypeptides derived from BLASTX were predicted using SWISS-MODEL (https://swissmodel.expasy.org/ (accessed on 3 March 2020)) and presented on the left-hand side in the above figure.

**Figure 5:**
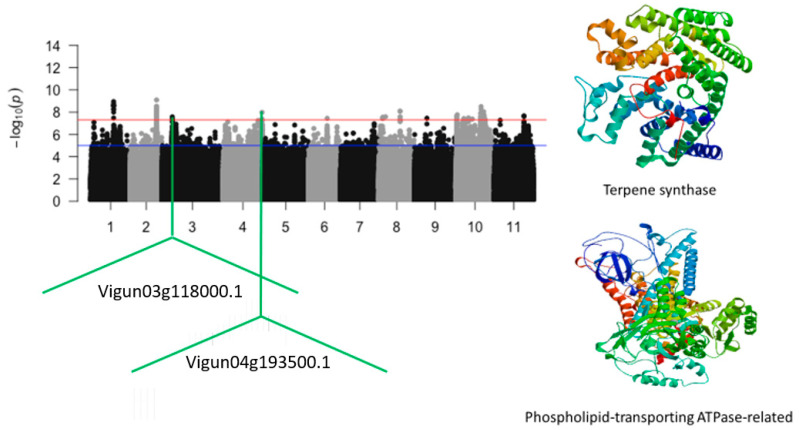
Manhattan plot relative tolerance index for leaf SPAD chlorophyll for salt stress in cowpea. The solid black and grey dots represent the SNPs. The *x*-axis is the chromosome number, and the *y*-axis is the LOD or −log10 of the *p*-value. The horizontal red and blue bars are two different LOD thresholds. Below the Manhattan plot are gene IDs from phytozome v.13 (https://phytozome.jgi.doe.gov/pz/portal.html (accessed on 3 March 2020)) corresponding to the significant locus on chromosomes 3 and 4. Codifying sequences of the gene IDs whose functions were related to drought stress were extracted and converted to amino acid sequences using BLASTX (https://blast.ncbi.nlm.nih.gov/Blast.cgi (accessed on 3 March 2020)). Tertiary structures of the proteins/polypeptides derived from BLASTX were predicted using SWISS-MODEL (https://swissmodel.expasy.org/ (accessed on 3 March 2020)) and presented on the left-hand side in the above figure.

**Figure 6:**
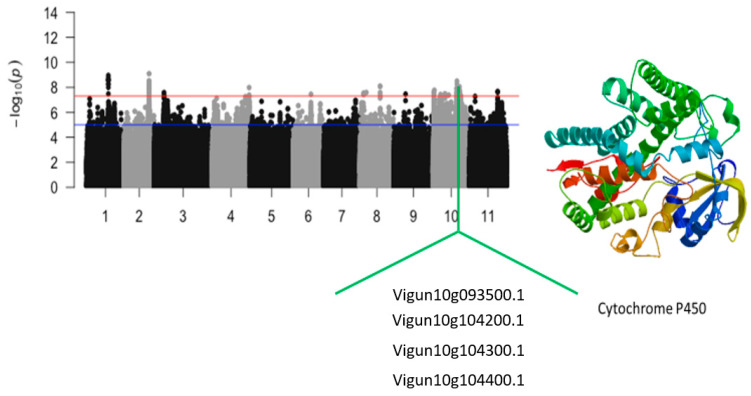
Manhattan plot relative tolerance index for leaf SPAD chlorophyll for salt stress in cowpea. The solid black and grey dots represent the SNPs. The *x*-axis is the chromosome number, and the *y*-axis is the LOD or −log10 of the *p*-value. The horizontal red and blue bars are two different LOD thresholds. Below the Manhattan plot are gene IDs from phytozome v.13 (https://phytozome.jgi.doe.gov/pz/portal.html (accessed on 3 March 2020)) corresponding to the significant locus on chromosome 8. Codifying sequences of the gene IDs whose functions were related to drought stress were extracted and converted to amino acid sequences using BLASTX (https://blast.ncbi.nlm.nih.gov/Blast.cgi (accessed on 3 March 2020)). Tertiary structures of the proteins/polypeptides derived from BLASTX were predicted using SWISS-MODEL (https://swissmodel.expasy.org/ (accessed on 3 March 2020)) and presented on the left-hand side in the above figure.

A total of 60 SNPs were found to be significantly associated with the relative tolerance index for chlorophyll content in cowpea (Table S2). These SNPs were identified on chromosomes 1, 2, 3, 4, 8, 10, and 11 (Figure 3, Figure 4, Figure 5 and Figure 6). The number of significant SNPs was 10, 21, 1, 1, 5, 20, and 2 on chromosomes 1, 2, 3, 4, 8, 10, and 11, respectively. LOD (−log_10_(*p*-value)) values ranged between 7.53 and 9.09. Three significant loci were found on chromosomes 1, 2, and 10. The significant SNPs that were mapped on chromosome 1 were Vu01_24245081 (LOD = 8.56), Vu01_24246312 (LOD = 8.56), Vu01_24246319 (LOD = 8.56), Vu01_24246550 (LOD = 8.26), Vu01_24246587 (LOD = 8.60), Vu01_24246822 (LOD = 8.95), Vu01_24246905 (LOD = 8.77), Vu01_24246981 (LOD = 8.64), Vu01_24248242 (LOD = 8.26), and Vu01_24249542 (LOD = 8.00). The SNP Vu01_24246822 was found within the structure of *Vigun01g086000.1*, which encoded for the GATA transcription factor (Figure 3). 

An additional significant locus was found to be associated with the relative tolerance index for chlorophyll. This locus was mapped on a 51 kb genomic region of chromosome 2 and defined by a total of 21 significant SNPs. This genomic region was gene-dense since a total of seven annotated genes were identified in this locus (Figure 4). The SNPs with the highest LOD values were within this region were Vu02_28094085 (LOD = 9.09), Vu02_28084764 (LOD = 8.53), Vu02_28105724 (LOD = 8.53), Vu02_28105725 (LOD = 8.33), Vu02_28075602 (LOD = 8.10), Vu02_28075604 (LOD = 8.10), Vu02_28112822 (LOD = 7.98), Vu02_28112832 (LOD = 7.98), Vu02_28071778 (LOD = 7.89), Vu02_28091358 (LOD = 7.78), Vu02_28111614 (LOD = 7.77), and Vu02_28108896 (LOD = 7.69). The following candidate genes consisting of *Vigun02g129000.1*, *Vigun02g129100.1*, *Vigun02g129200.1*, *Vigun02g129300.1*, and *Vigun02g129400.1*, were found close to the SNP location (Table 2). These candidate genes were a cluster of NAD-dependent epimerase/dehydratase (Figure 4).

The significant SNPs that were identified on chromosomes 3 and 4 were Vu03_10976477 (LOD = 7.58) and Vu04_41756724 (LOD = 7.98), respectively. The SNPs were in the vicinity of *Vigun03g118000.1* and *Vigun04g193500.1*, encoding for terpene synthase and phospholipid-transporting ATPase, respectively (Figure 5). The significant SNPs that were located on chromosome 8 were Vu08_4118979 (LOD = 7.54), Vu08_7137752 (LOD = 7.58), Vu08_22719007 (LOD = 8.08), Vu08_22719008 (LOD = 8.08), and Vu08_22719042 (LOD = 7.58). However, no annotated genes were found in the vicinity of these SNPs. An 86 kb region of chromosome 10 could also be a good candidate locus for the relative tolerance index for chlorophyll content under salt stress in cowpea. This region was defined by a total of eight significant SNPs. These SNPs consisted of Vu10_29847718 (LOD = 7.55), Vu10_29848338 (LOD = 7.59), Vu10_29864524 (LOD = 7.78), Vu10_29864555 (LOD = 7.67), Vu10_29864829 (LOD = 7.78), Vu10_29865036 (LOD = 8.04), Vu10_29933934 (LOD = 7.63), and Vu10_29933946 (LOD = 7.63). In addition, this region harbored a cluster of cytochrome P450 (Figure 6).

### 2.3. Leaf Injury Score under Salt Stress

**Figure 7:**
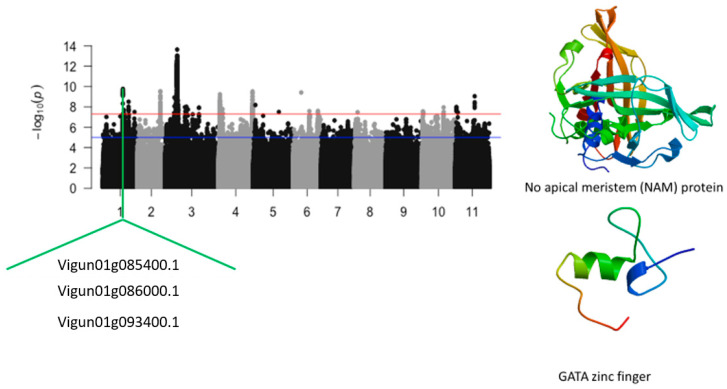
Manhattan plot for tolerance to leaf injury score under salt stress in cowpea. The solid black and grey dots represent the SNPs. The *x*-axis is the chromosome number, and the *y*-axis is the LOD or −log10 of the *p*-value. The horizontal red and blue bars are two different LOD thresholds. Below the Manhattan plot are gene IDs from phytozome v.13 (https://phytozome.jgi.doe.gov/pz/portal.html (accessed on 3 March 2020)) corresponding to the significant locus on chromosome 1. Codifying sequences of the gene IDs whose functions were related to drought stress were extracted and converted to amino acid sequences using BLASTX (https://blast.ncbi.nlm.nih.gov/Blast.cgi (accessed on 3 March 2020)). Tertiary structures of the proteins/polypeptides derived from BLASTX were predicted using SWISS-MODEL (https://swissmodel.expasy.org/ (accessed on 3 March 2020)) and presented on the left-hand side in the above figure.

**Figure 8:**
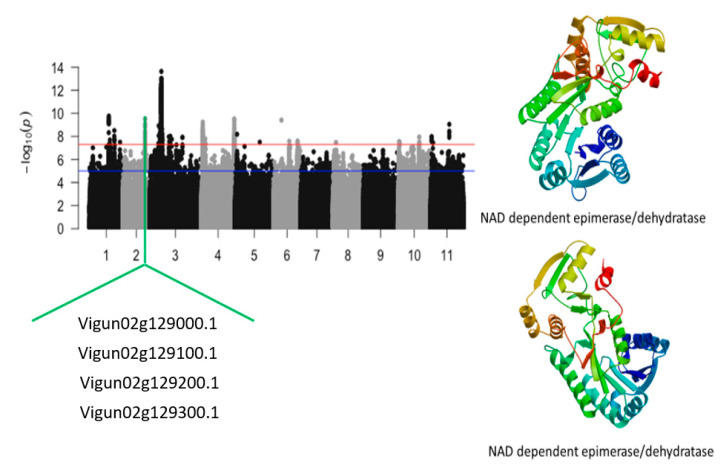
Manhattan plot for tolerance to leaf injury score under salt stress in cowpea. The solid black and grey dots represent the SNPs. The *x*-axis is the chromosome number, and the *y*-axis is the LOD or −log10 of the *p*-value. The horizontal red and blue bars are two different LOD thresholds. Below the Manhattan plot are gene IDs from phytozome v.13 (https://phytozome.jgi.doe.gov/pz/portal.html (accessed on 3 March 2020)) corresponding to the significant locus on chromosome 2. Codifying sequences of the gene IDs whose functions were related to drought stress were extracted and converted to amino acid sequences using BLASTX (https://blast.ncbi.nlm.nih.gov/Blast.cgi (accessed on 3 March 2020)). Tertiary structures of the proteins/polypeptides derived from BLASTX were predicted using SWISS-MODEL (https://swissmodel.expasy.org/ (accessed on 3 March 2020)) and presented on the left-hand side in the above figure.

**Figure 9:**
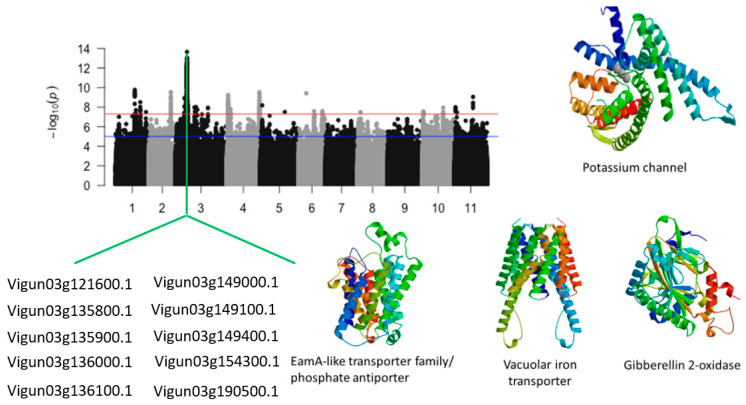
Manhattan plot for tolerance to leaf injury score under salt stress in cowpea. The solid black and grey dots represent the SNPs. The *x*-axis is the chromosome number, and the *y*-axis is the LOD or −log10 of the *p*-value. The horizontal red and blue bars are two different LOD thresholds. Below the Manhattan plot are gene IDs from phytozome v.13 (https://phytozome.jgi.doe.gov/pz/portal.html (accessed on 3 March 2020)) corresponding to the significant locus on chromosome 3. Codifying sequences of the gene IDs whose functions were related to drought stress were extracted and converted to amino acid sequences using BLASTX (https://blast.ncbi.nlm.nih.gov/Blast.cgi (accessed on 3 March 2020)). Tertiary structures of the proteins/polypeptides derived from BLASTX were predicted using SWISS-MODEL (https://swissmodel.expasy.org/ (accessed on 3 March 2020)) and presented on the left-hand side in the above figure.

**Figure 10:**
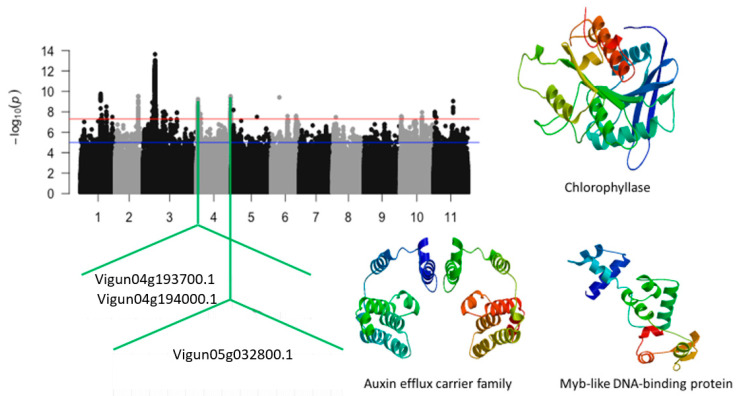
Manhattan plot for tolerance to leaf injury score under salt stress in cowpea. The solid black and grey dots represent the SNPs. The *x*-axis is the chromosome number, and the *y*-axis is the LOD or −log10 of the *p*-value. The horizontal red and blue bars are two different LOD thresholds. Below the Manhattan plot are gene IDs from phytozome v.13 (https://phytozome.jgi.doe.gov/pz/portal.html (accessed on 3 March 2020)) corresponding to the significant locus on chromosome 4. Codifying sequences of the gene IDs whose functions were related to drought stress were extracted and converted to amino acid sequences using BLASTX (https://blast.ncbi.nlm.nih.gov/Blast.cgi (accessed on 3 March 2020)). Tertiary structures of the proteins/polypeptides derived from BLASTX were predicted using SWISS-MODEL (https://swissmodel.expasy.org/ (accessed on 3 March 2020)) and presented on the left-hand side in the above figure.

A total of 1667 SNPs were found to be significantly associated with leaf injury score under salt stress in cowpea. These significant SNPs were located on chromosomes 1, 2, 3, 4, 5, 3, 10, and 11 (Figure 7, Figure 8, Figure 9 and Figure 10). The number of SNP was 18, 53, 1494, 84, 1, 3, 3, and 11 on chromosomes 1, 2, 3, 4, 5, 3, 10, and 11, respectively. LOD (−log_10_(*p*-value)) values varied from 7.52 to 13.63. The first significant locus associated with leaf injury score was a 140 kb region of chromosome 1. This genomic region contained the SNPs Vu01_24112868 (LOD = 8.33), Vu01_24245081 (LOD = 9.30), Vu01_24246312 (LOD = 9.44), Vu01_24246319 (LOD = 9.44), Vu01_24246550 (LOD = 9.23), Vu01_24246587 (LOD = 9.28), Vu01_24246822 (LOD = 9.64), Vu01_24246905 (LOD = 9.76), Vu01_24246981 (LOD = 9.58), Vu01_24248242 (LOD = 9.11), and Vu01_24249542 (LOD = 9.08). Two annotated genes, *Vigun01g085400.1* and *Vigun01g086000.1*, having functional annotations relevant to plant physiology, were identified in this region (Figure 7). An additional significant SNP, Vu01_25586428 (LOD = 7.69), mapped at more than 1 Mb of the 140 kb locus, was located in the vicinity of *Vigun01g093400.1*, encoding for plasma-membrane choline transporter. A cluster of significant SNPs (Vu01_31228168 (LOD = 8.02), Vu01_31228899 (LOD = 7.77), Vu01_31228901 (LOD = 7.77), Vu01_31228974 (LOD = 7.59), Vu01_31228996 (LOD = 7.64), and Vu01_31229389 (LOD = 8.51)) located towards the end of chromosome 1 were also identified. However, no annotated genes were found in the vicinity of this cluster.

A group of 53 significant SNPs, mapped on a 68 kb region of chromosome 2, was also identified. The SNPs with the highest LOD values in this region were Vu02_28050011 (LOD = 9.53), Vu02_28054154 (LOD = 9.48), Vu02_28105724 (LOD = 9.09), Vu02_28105725 (LOD = 9.09), Vu02_28090457 (LOD = 8.94), Vu02_28050187 (LOD = 8.79), Vu02_28064123 (LOD = 8.46), Vu02_28090387 (LOD = 8.44), Vu02_28094085 (LOD = 8.37), Vu02_28084764 (LOD = 8.27), Vu02_28064103 (LOD = 8.26), Vu02_28050297 (LOD = 8.22), Vu02_28060786 (LOD = 8.20), Vu02_28091358 (LOD = 8.19), and Vu02_28068945 (LOD = 8.17). The 68 kb of chromosome 2 harbored significant clusters of NAD-dependent epimerase/dehydratase (Figure 8). 

Chromosome 3 harbored the most important significant locus associated with tolerance to leaf score injury under salt stress in cowpea (Figure 9). This locus was a 1.5 Mb region of chromosome 3 and harbored more than 1400 significant SNPs. The SNPs with the highest LOD values in this region were Vu03_14737814 (LOD = 13.63), Vu03_14726223 (LOD = 13.04), Vu03_14719792 (LOD = 13.01), Vu03_14737840 (LOD = 12.98), Vu03_14716271 (LOD = 12.94), Vu03_14714710 (LOD = 12.88), Vu03_14722481 (LOD = 12.87), Vu03_14722442 (LOD = 12.86), Vu03_14737848 (LOD = 12.65), Vu03_14725396 (LOD = 12.63), Vu03_14722398 (LOD = 12.58), Vu03_14734685 (LOD = 12.58), Vu03_14726150 (LOD = 12.54), and Vu03_14720653 (LOD = 12.52). Several annotated genes were found within the 1.5 Mb region of chromosome 3. The GWAS signal peak in this region was within the structure of a potassium channel (*Vigun03g144700.1*) (Figure 9) (Table 1). In addition, biomolecule transporters (iron transporters, phosphate transporters…) such as *Vigun03g135800.1*, *Vigun03g135900.1*, *Vigun03g136000.1*, *Vigun03g136300.1*, and *Vigun03g136400.1* were found to be in the vicinity of the significant SNPs.

Significant GWAS peaks were also identified on chromosome 4. The SNPs that were closest to annotated genes were Vu04_1785520 (LOD = 8.55), Vu04_1801689 (LOD = 8.32), Vu04_1857562 (LOD = 8.14), Vu04_1876606 (LOD = 7.52), Vu04_1896799 (LOD = 8.49), Vu04_1916362 (LOD = 9.01), Vu04_2001620 (LOD = 8.23), Vu04_2535911 (LOD = 7.90), Vu04_5101729 (LOD = 7.78), Vu04_41757989 (LOD = 8.30), Vu04_41787263 (LOD = 7.95), Vu04_41800162 (LOD = 8.72), and Vu04_41850683 (LOD = 7.67) (Table S3). The annotated genes having functional annotations that were most relevant to tolerance to plant abiotic stress were *Vigun04g025900.1*, *Vigun04g031500.1*, and *Vigun04g054000.1*. These annotated genes encode for chlorophyllase, auxin efflux carrier family, and Myb-like DNA binding protein, respectively (Figure 10). In addition, annotated genes involved in plant physiology were also identified. These genes consisted of *Vigun04g023800.1*, *Vigun04g193600.1*, *Vigun04g193700.1*, *Vigun04g194000.1*, and *Vigun04g194100.1*.

### 2.4. Protein Homologs and Gene Ontology

Protein homolog search was investigated for the candidate genes with functional annotations that could be linked to tolerance to plant abiotic stress. In this study, the search was carried out across the genomes of legumes such as soybean, common bean, and Medicago. Proteins that have a similarity >90% with the query were taken into account. In order to estimate the number of copies of each candidate gene for cowpea, a search was conducted within the cowpea genome. For the candidate genes associated with leaf SPAD chlorophyll under salt stress, multiple copies of *Vigun02g128900.1*, *Vigun02g129000.1*, and *Vigun02g129300.1* within the cowpea genome (Table 2). The candidate genes *Vigun01g086000.1*, *Vigun02g128700.1*, *Vigun02g129100.1*, *Vigun02g129200.1*, *Vigun02g129400.1*, and *Vigun02g129500*.1 had one to three copies within the cowpea genome. The number of protein homologs was highest within the soybean genome, whereas it was lowest within the Medicago genome (Table 2). For the candidate genes associated with relative tolerance index for chlorophyll, a large number of copies of *Vigun10g104200.1*, *Vigun10g104300.1*, and *Vigun10g104400.1* were found within the cowpea genome. The candidate gene *Vigun04g193500.1* was unique within the cowpea genome. The candidate genes *Vigun01g086000.1*, *Vigun02g129000.1*, *Vigun02g129100.1*, *Vigun02g129200.1*, *Vigun02g129300.1*, *Vigun02g129400.1*, *Vigun03g118000.1*, and *Vigun10g093500.1* had one to four copies within the cowpea genome. Overall, the number of homologs between common bean and cowpea was very close. Among the four legume species compared in this study, the soybean genome had the largest number of copies. For the candidate genes associated with leaf injury score, the number of gene duplications is less significant compared to other traits. The candidate genes *Vigun01g086000.1*, *Vigun03g144700.1*, *Vigun04g025900.1*, and *Vigun04g193700.1* were unique within the cowpea genome. The candidate genes *Vigun04g193700.1*, *Vigun02g129000.1*, *Vigun02g129200.1*, *Vigun03g135800.1*, *Vigun03g136300.1*, *Vigun03g149400.1*, and *Vigun04g054000.1* had one to four copies within the cowpea genome. Vigun03g135800.1 seemed to be abundant within the soybean, common bean, and Medicago genomes. However, only one of the common bean genomes had a single copy of *Vigun01g086000.1*.

### 2.5. Overlapping SNPs and Functional Annotations

**Figure 11:**
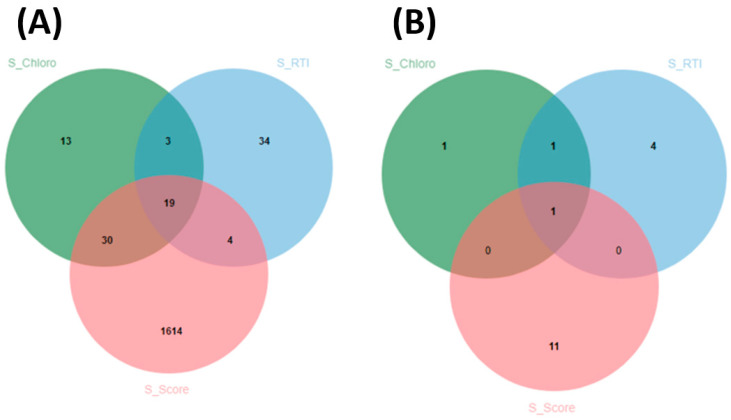
(**A**) Venn diagram showing the number of overlapping significant SNPs associated with leaf SPAD chlorophyll under salt stress (S_Chloro), relative tolerance index for leaf SPAD chlorophyll (S_RTI), and leaf injury score under salt stress (S_Score) in cowpea. (**B**) Venn diagram showing the number of unique functional annotations for candidate genes associated with leaf SPAD chlorophyll under salt stress (S_Chloro), relative tolerance index for leaf SPAD chlorophyll (S_RTI), and leaf injury score under salt stress (S_Score) in cowpea. Venn diagrams were established using http://jvenn.toulouse.inra.fr/app/example.html (accessed on 10 March 2020).

The number of overlapping SNPs between traits was visualized using a Venn diagram (Figure 11A). On the Venn diagram, the significant SNPs associated with leaf SPAD chlorophyll under salt stress, relative tolerance index for chlorophyll, and leaf score injury were represented using solid green, blue, and pink circles, respectively (Figure 11A). The number of SNPs associated with leaf SPAD chlorophyll under salt stress, relative tolerance index for chlorophyll, and leaf score injury were 65, 60, and 1667, respectively. 

A total of 19 SNPs overlapped between leaf SPAD chlorophyll under salt stress, relative tolerance index for chlorophyll, and leaf score injury, as shown in Figure 11A, suggesting that there could be a common genetic mechanism controlling these traits. The number of common SNPs between leaf SPAD chlorophyll under salt stress and tolerance index for chlorophyll was 3. The number of overlapping SNPs between the relative tolerance index for chlorophyll and leaf injury score was 4. The number of shared SNPs between leaf SPAD chlorophyll under salt stress and leaf injury score was 30. These results provided strong evidence of the interdependency between these traits at the genetic level.

Overlapping functional annotations between candidate genes associated with leaf SPAD chlorophyll under salt stress, relative tolerance index for chlorophyll, and leaf injury score were also visualized using a Venn diagram (Figure 11B). Duplicated functional annotation names were removed, and only the number of unique names was displayed on the Venn diagram. Color coding was similar to Figure 11A. The three traits investigated for salt tolerance showed a common functional annotation, supporting the evidence of the potential common genetic mechanism controlling these traits (Figure 11). In addition, a common functional annotation was identified for the candidate genes associated with leaf SPAD chlorophyll under salt stress and relative tolerance index for salt stress. No common functional annotation was found between the candidate genes associated with leaf SPAD chlorophyll and leaf injury score under salt stress. Similar results were found in the candidate genes associated with relative tolerance for chlorophyll and leaf injury score under salt stress.

## 3. Discussion

Whole genome resequencing has been more and more popular in plant genetic-related studies. It allows for the discovery of a large number of SNPs that can be used in GWAS. Thanks to the large number of SNPs, the likelihood of discovering good candidate genes is higher [26,27]. This study was one of the earliest reports in cowpea using a whole genome resequencing data to conduct GWAS for salt tolerance in cowpea. Whole genome resequencing provided a total of 14,465,516 SNPs. GWAS was conducted using a total of 5,884,299 filtered and high-quality SNPs. 

In this study, a total of 65, 60, and 1667 SNPs were found to be significantly associated with leaf SPAD chlorophyll under salt stress, relative tolerance index for chlorophyll, and leaf score injury, respectively. The first reported molecular markers associated with salt tolerance in cowpea were Scaffold87490_622, Scaffold87490_630, C35017374_128, Scaffold93827_270, Scaffold68489_600, Scaffold87490_633, Scaffold87490_640, Scaffold82042_3387, C35069468_1916, and Scaffold93942_1089 [24]. These are SNP markers that were obtained from genotyping-by-sequencing. The sequence that contains these SNPs was realigned to the cowpea genome to find whether they are mapped in the vicinity of the SNPs reported in this paper. The SNPs Scaffold87490_622, Scaffold87490_633, and C35069468_1916 were found at about 10 kb downstream of Vu01_24246905, indicating that the results from this study are complementary with the GBS study that was previously reported.

One of the most interesting findings was the discovery of a strong GWAS signal that was mapped on a 1 Mb region of chromosome 3, which was associated with tolerance to leaf score injury under salt stress. The peak of this signal corresponded to *Vigun03g144700.1*, which encodes for a potassium channel. This potassium channel has been described to be activated upon salt stress in cowpea in order to enhance the transport of K^+^ under salt stress in cowpea [28]. Previous investigations have shown that salt-tolerant cowpea had a higher K^+^/Na^+^ ratio in leaves [16,29]. Therefore, the GWAS approach we used in this study has successfully targeted a gene that is involved in salt tolerance in cowpea. In addition, our previous research revealed a K^+^ channel protein being involved in salt tolerance in a MAGIC population, which is in agreement with the GWAS result in this study. Potassium channel proteins have also been well-described for enhancing tolerance to salinity in other species. K^+^ channel-related genes were shown to be upregulated under salt stress in tomato and soybean [30]. 

Genes encoding for NAD-dependent dehydratase have also been found in the vicinity of the significant SNPs associated with salt tolerance. These genes have been demonstrated to regulate stress in rice [31]. A gene encoding for auxin efflux carrier was also found within the GWAS peaks. Auxin efflux proteins were reported to have a significant role in assisting Arabidopsis thaliana with regulating salt stress [32]. The auxin efflux carriers regulate the variation in auxin flow during salt stress and are also involved in regulating meristem size for plants under salt stress. Results also indicated the involvement of a chlorophyllase gene in salt tolerance. However, there is no report yet highlighting the role of chlorophyllase in salt tolerance. We would suggest that chlorophyllase is a salt-susceptible gene since it is involved in chlorophyll degradation [33]. Genes involved in vacuolar iron transporters were also identified. Our previous investigation on salt tolerance identified these genes in a MAGIC population. These transporters are also involved in salt tolerance [34]. In soybean, the Na^+^/H^+^ antiporter gene, *GmCHX1*, has been well described in conferring salt tolerance [35]. A simple BLAST search showed that an orthologue of this gene can be found on chromosome 7 of cowpea. However, no strong GWAS peak was found on this chromosome. We could assume that this gene might be associated with a rare allele so that our GWAS approach failed to identify it.

A large number of molecular markers that are associated with cowpea salt tolerance have been identified in this study. A SNP validation is required prior to using these markers in a breeding program for Marker-Assisted Selection (MAS). The results from this investigation also contributed to a better understanding of the genetics of salt tolerance mechanism in cowpea. The candidate genes that were relevant to salt tolerance will be validated in further studies. Conducting the salt tolerance under greenhouse conditions could be a limitation of this study. However, to date, greenhouse phenotyping remains the most affordable and accurate way to evaluate salt tolerance since a lot of uncontrolled factors can occur during field screening. Therefore, repeating the experiments under field conditions could be a major challenge.

## 4. Materials and Methods

### 4.1. Plant Materials and Phenotyping

A total of 331 cowpea genotypes were evaluated for salt tolerance. This association panel consisted of breeding lines from the University of Arkansas, Fayetteville, and the University of California, Riverside [36], and Plant Introductions (PIs) from the U.S. Department of Agriculture (USDA) Germplasm Resources Information Network (GRIN) cowpea accessions and provided by the USDA Plant Genetic Resources Conservation Unit at Griffin, GA, USA. 

Phenotypic data were collected in our previous project [25]. Salt tolerance evaluation was conducted under greenhouse conditions at the University of Arkansas, Fayetteville, with average day/light temperatures of 26 °C/21 °C and an average daylight length of 14 hours. Salt tolerance was performed using a previously described methodology [37]. Briefly, a total of four cowpea seedlings were established in pots filled with 100 g Sunshine Natural & Organic (Agawam, MA, USA). The experiments were conducted using a randomized complete block design (RCBD) with 2 replications within each block. A total of 4 blocks were used. Each pot corresponded to one replication. For each genotype, one pot was subjected to salt treatment, whereas another one was irrigated with deionized water and used as a control. A total of 12 pots were established on a rectangular plastic tray to facilitate irrigation. Salt treatment (NaCl) began at the first trifoliate leaf stage (V1 stage) [38] and was conducted by supplying a solution of 200 mM NaCl to each rectangular plastic tray [37]. Two-thirds of pot’s height was fully soaked with either deionized water or salt solution during irrigation [37]. A salt-tolerant genotype (‘09-529’) and a salt-susceptible genotype (PI255774) [37] were used as checks. Data measurements were previously described, and the phenotypic data used for this study are from our previous report [25]. From our previous phenotyping efforts, we found leaf injury score ranged between 1.4 to 9, leaf SPAD chlorophyll under salt stress varied from 6.4 to 39.9, and relative tolerance index for chlorophyll content ranged between 16.7 to 121.0. These phenotypic data indicate that a greater variability in salt tolerance traits exist among the cowpea accessions used in this study.

### 4.2. Genotyping

#### 4.2.1. DNA Extraction, Library Preparation, and Whole-Genome Resequencing

Young cowpea leaves were harvested from one plant, and all seeds used during the experiments were derived from that one plant. Genomic DNA was extracted from freeze-dried young cowpea leaves using the CTAB (hexadecyltrimethyl ammonium bromide) protocol [39]. Leaves were ground using a Mixer Mill MM 400^®^ (Haan, Germany). DNA buffer was added to each sample. The mixture DNA buffer sample was centrifuged at 13,000 rpm for 10 min. Proteins were denatured by adding a solution of 1 mL of chloroform–isoamyl alcohol (24:1) to each sample. The addition of 1 mL of isopropanol helped DNA precipitate. In order to optimize DNA precipitation, samples were stored at −20 °C overnight. DNA pellets were washed using 70% and 90% ethanol. Washed DNA pellets were air dried. RNA was removed by adding 3 µl of RNAse to each sample. DNA was kept in a solution of 200 µL of 0.1× TE. DNA was quantity using a NanoDrop 200c spectrophotometer (Thermo SCIENTIFIC, Wilmington, DE, USA) and quality-checked on a 1%-agarose gel with ethidium bromide stain. 

DNA sequencing was conducted by Novogene (http://en.novogene.com/ (accessed on 2 February 2020)). DNA was cleaved in 350-pb fragments using Covaris S2^®^ (Covaris, Inc., Woburn, MA, USA). DNA library involved the sheared DNA fragments NEBNext DNA Library Prep Reagent Set for Illumina (BioLabs, Inc., Ipswich, MA, USA). DNA fragments were end-repaired. Poly-A tails were added to each fragment. In situ PCR amplification was conducted as previously described [40]. Sequencing was performed using Illumina HiSeq X Ten Series (http://www.illumina.com/systems/hiseq-x-sequencing-system/system.html (accessed on 17 January 2020)) with an average of 10× coverage.

#### 4.2.2. SNP Calling, Mapping, and Filtering

Reads were aligned to the cowpea reference genome [41] using SOAPaligner/soap2 (http://soap.genomics.org.cn/ (accessed on 17 January 2020)). SNP calling was conducted using SOAPsnp v 1.05 [42]. Accessions with more than 20% missing data were removed. Triallelic SNPs and those with more than 20% missing data were also removed. SNPs with a heterozygosity greater than 20% were removed as well. The minor allele frequency (MAF) threshold was 5%. GWAS was conducted using filtered SNPs.

### 4.3. Genome-Wide Association Study (GWAS)

GWAS was performed using Bayesian Information and Linkage Disequilibrium Iteratively Nested Keyway (BLINK) model [43]. BLINK has been demonstrated to be statistically more powerful than the previously developed models [44]. SNP was significant when above the FDR-adjusted threshold and computed in R (*p* < 3 × 10^−8^). BLINK model was built upon the Fixed and Random Model Circulating Probability Unification (FarmCPU) model. In FarmCPU, markers were assumed to be evenly distributed across the genome, which was not necessarily true. BLINK used the LD information to relax this assumption. In addition, FarmCPU could be computationally intensive due to the random model part of its algorithm. The random model was replaced by a fixed model in BLINK. The two fixed effect models in BLINK are described below.

(1) $$FEM\;(1)\;y_{i}=M_{i1}b_{1}+M_{i2}b_{2}+...+M_{ik}b_{k}+M_{ij}d_{j}+e_{i}\;$$

(2) $$FEM\;(2)\;y_{i}=M_{i1}b_{1}+M_{i2}b_{2}+...+M_{ij}d_{j}+e_{i}$$

with y_i_ being the vector phenotype, M_i1_, M_i2_b_2_, …, M_ik_ the genotypes of k pseudo QTNs that were initially empty and with effects b_1_, b_2_, …, b_k_, respectively, M_ij_ being the j^th^ genetic marker of the i^th^ sample, and e_i_ being the residual having a distribution with mean zero and a variance σ^2^_e_. Overlapping SNP markers between different traits were visualized using a Venn diagram and designed using the online software program accessible at http://jvenn.toulouse.inra.fr/app/example.html (accessed on 10 March 2020).

### 4.4. Candidate Gene Search and Synteny Analysis

By taking into account the number of SNPs involved in this study, the genome size of cowpea, and the average length of a gene within the cowpea genome, we investigated the annotated genes within 10 kb genomic region flanking an SNP using Phytozome v.13 (https://phytozome.jgi.doe.gov/pz/portal.html (accessed on 2 March 2020)). We considered annotated genes that were involved in plant physiology and/or tolerance to abiotic stress. Functional annotations of each annotated gene were obtained using Phytozome v. 13 (https://phytozome.jgi.doe.gov/pz/portal.html (accessed on 2 March 2020)). Coding sequences of the annotated genes relevant to plant physiology and/or tolerance to abiotic stress were extracted. The extracted sequences were used as query to perform BLASTx (https://blast.ncbi.nlm.nih.gov/Blast.cgi (accessed on 2 March 2020)) in order to obtain the amino acid sequence. Protein homolog search in other legumes such as soybean, common bean, and *Medicago truncatula* Gaertn was performed using the amino acid sequence. Only hits with similarity greater than 90% were considered. The tertiary structure of the amino acid sequence was predicted using SWISS-MODEL (https://swissmodel.expasy.org/ (accessed on 2 March 2020)).

## 5. Conclusions

In this study, strong GWAS peaks associated with leaf SPAD chlorophyll under salt stress, relative tolerance index for chlorophyll, and tolerance to leaf injury score under salt stress were identified. A total of 65, 60, and 1667 significant SNPs were found to be associated with leaf SPAD chlorophyll under salt stress, relative tolerance index for chlorophyll, and tolerance to leaf injury score under salt stress, respectively. Leaf SPAD chlorophyll under salt stress was characterized by a strong candidate locus by an 890 kb region of chromosome 2. Two candidate loci were found to be associated with the relative tolerance index for chlorophyll and mapped on chromosomes 1 and 2. A strong candidate locus defined by a 1-Mb region of chromosome 3 was associated with tolerance to leaf injury score under salt stress in cowpea. The results from this study could be used in cowpea breeding through Marker-Assisted Selection (MAS). To the best of our knowledge, this is the first report on cowpea GWAS using whole genome resequencing data.

## Figures and Tables

**Table 1:** List of significant SNPs close to candidate genes and associated with leaf SPAD chlorophyll under salt stress, relative tolerance index for chlorophyll, and leaf injury score under salt stress in cowpea. SNP, CHR, BP, Pval, and LOD refers to SNP_ID, chromosome number, physical location (in bp), *p*-value, and -log10 of p-value (LOD), respectively. Gene_ID and functional annotations were obtained from Pythozome v.13.

Traits	SNP	CHR	BP	Pval	LOD	Gene_ID	Functional_Annotation
Leaf SPAD chlorophyll under salt treatment	Vu01_24245081	1	24245081	2.70 × 10^−8^	7.57	*Vigun01g086000.1*	GATA transcription factor
	Vu02_28035590	2	28035590	4.73 × 10^−9^	8.33	*Vigun02g128700.1*	Inorganic phosphatase
	Vu02_28044965	2	28044965	3.71 × 10^−10^	9.43	*Vigun02g128800.1*	Replication factor C
	Vu02_28050297	2	28050297	3.54 × 10^−11^	10.45	*Vigun02g128900.1*	NAD dependent epimerase/dehydratase
	Vu02_28054154	2	28054154	2.09 × 10^−11^	10.68	*Vigun02g129000.1*	NAD dependent epimerase/dehydratase
	Vu02_28064103	2	28064103	9.85 × 10^−10^	9.01	*Vigun02g129100.1*	NAD dependent epimerase/dehydratase
	Vu02_28068945	2	28068945	2.47 × 10^−10^	9.61	*Vigun02g129200.1*	NAD dependent epimerase/dehydratase
	Vu02_28084764	2	28084764	2.34 × 10^−10^	9.63	*Vigun02g129300.1*	NAD dependent epimerase/dehydratase
	Vu02_28090457	2	28090457	5.66 × 10^−10^	9.25	*Vigun02g129400.1*	NAD dependent epimerase/dehydratase
	Vu02_28105724	2	28105724	8.96 × 10^−11^	10.05	*Vigun02g129500.1*	NAD dependent epimerase/dehydratase
Relative tolerance index for chlorophyll	Vu01_24246822	1	24246822	1.14 × 10^−9^	8.95	*Vigun01g086000.1*	GATA transcription factor
	Vu02_28061740	2	28061740	2.28 × 10^−8^	7.64	*Vigun02g129000.1*	NAD dependent epimerase/dehydratase
	Vu02_28071778	2	28071778	1.30 × 10^−8^	7.89	*Vigun02g129100.1*	NAD dependent epimerase/dehydratase
	Vu02_28084764	2	28084764	2.96 × 10^−9^	8.53	*Vigun02g129200.1*	NAD dependent epimerase/dehydratase
	Vu02_28105725	2	28105725	4.70 × 10^−9^	8.33	*Vigun02g129300.1*	NAD dependent epimerase/dehydratase
	Vu02_28112832	2	28112832	1.06 × 10^−8^	7.98	*Vigun02g129400.1*	NAD dependent epimerase/dehydratase
	Vu03_10976477	3	10976477	2.64 × 10^−8^	7.58	*Vigun03g118000.1*	Terpene synthase
	Vu04_41756724	4	41756724	1.05 × 10^−8^	7.98	*Vigun04g193500.1*	Phospholipid-transporting ATPase-related
	Vu10_27003173	10	27003173	1.93 × 10^−8^	7.72	*Vigun10g093500.1*	Xanthoxin dehydrogenase/Abscisic acid biosynthesis
	Vu10_29847718	10	29847718	2.79 × 10^−8^	7.55	*Vigun10g104200.1*	Cytochrome P450
	Vu10_29864524	10	29864524	1.67 × 10^−8^	7.78	*Vigun10g104300.1*	Cytochrome P450
	Vu10_29933934	10	29933934	2.37 × 10^−8^	7.63	*Vigun10g104400.1*	Cytochrome P450
Leaf injury score under salt stress	Vu01_24112868	1	24112868	4.68 × 10^−9^	8.33	*Vigun01g085400.1*	No apical meristem (NAM) protein
	Vu01_24249542	1	24249542	8.23 × 10^−10^	9.08	*Vigun01g086000.1*	GATA zinc finger
	Vu01_25586428	1	25586428	2.04 × 10^−8^	7.69	*Vigun01g093400.1*	Plasma-membrane choline transporter
	Vu02_28050011	2	28050011	2.95 × 10^−10^	9.53	*Vigun02g129000.1*	NAD dependent epimerase/dehydratase
	Vu02_28064123	2	28064123	3.44 × 10^−9^	8.46	*Vigun02g129100.1*	NAD dependent epimerase/dehydratase
	Vu02_28090457	2	28090457	1.14 × 10^−9^	8.94	*Vigun02g129200.1*	NAD dependent epimarase/dehydratase
	Vu02_28105725	2	28105725	8.07 × 10^−10^	9.09	*Vigun02g129300.1*	NAD dependent epimarase/dehydratase
	Vu03_11383713	3	11383713	1.20 × 10^−9^	8.92	*Vigun03g121600.1*	Malate dehydrogenase
	Vu03_13297388	3	13297388	9.20 × 10^−9^	8.04	*Vigun03g135800.1*	Vacuolar iron transporter
	Vu03_13305589	3	13305589	6.45 × 10^−9^	8.19	*Vigun03g135900.1*	Vacuolar iron transporter
	Vu03_13313938	3	13313938	8.57 × 10^−9^	8.07	*Vigun03g136000.1*	Vacuolar iron transporter
	Vu03_13334160	3	13334160	4.32 × 10^−9^	8.36	*Vigun03g136100.1*	Histidine decarboxylase
	Vu03_13357176	3	13357176	2.44 × 10^−9^	8.61	*Vigun03g136300.1*	EamA-like transporter family/phosphate antiporter
	Vu03_13363517	3	13363517	4.54 × 10^−9^	8.34	*Vigun03g136400.1*	EamA-like transporter family/phosphate antiporter
	Vu03_13509429	3	13509429	1.37 × 10^−8^	7.86	*Vigun03g137600.1*	tRNA-splicing endonuclease positive effector-related
	Vu03_14318570	3	14318570	3.03 × 10^−9^	8.52	*Vigun03g142100.1*	Tetrahydroberberine oxidase
	Vu03_14369744	3	14369744	1.20 × 10^−8^	7.92	*Vigun03g142200.1*	Tetrahydroberberine oxidase
	Vu03_14373278	3	14373278	1.75 × 10^−8^	7.76	*Vigun03g142300.1*	Tetrahydroberberine oxidase
	Vu03_14737814	3	14737814	2.33 × 10^−14^	13.63	*Vigun03g144700.1*	Potassium channel
	Vu03_14760979	3	14760979	8.65 × 10^−11^	10.06	*Vigun03g144800.1*	WRKY transcription factor
	Vu03_15238396	3	15238396	1.78 × 10^−8^	7.75	*Vigun03g148600.1*	Flavine reductase-related
	Vu03_15286489	3	15286489	2.79 × 10^−8^	7.55	*Vigun03g148900.1*	CCR4-NOT transcription complex subunit
	Vu03_15308668	3	15308668	5.92 × 10^−9^	8.23	*Vigun03g149000.1*	Eukaryotic cytochrome b561
	Vu03_15338189	3	15338189	5.92 × 10^−9^	8.23	*Vigun03g149100.1*	DNA-directed RNA polymerase II subunit RPB7
	Vu03_15380199	3	15380199	2.32 × 10^−9^	8.64	*Vigun03g149400.1*	Gibberellin 2-oxidase
	Vu03_16376823	3	16376823	1.95 × 10^−8^	7.71	*Vigun03g154300.1*	Leucine-rich repeat protein
	Vu03_26130498	3	26130498	9.70 × 10^−9^	8.01	*Vigun03g190500.1*	Polysaccharide biosynthesis
	Vu04_1785520	4	1785520	2.83 × 10^−9^	8.55	*Vigun04g023800.1*	Zinc finger protein-like protein
	Vu04_1801689	4	1801689	4.76 × 10^−9^	8.32	*Vigun04g023900.1*	Core-2/I-Branching enzyme
	Vu04_1857562	4	1857562	7.27 × 10^−9^	8.14	*Vigun04g024100.1*	Calmodulin binding protein
	Vu04_1876606	4	1876606	2.99 × 10^−8^	7.52	*Vigun04g024200.1*	Protein kinase family
	Vu04_1896799	4	1896799	3.20 × 10^−9^	8.49	*Vigun04g024700.1*	Protein tyrosin kinase
	Vu04_1916362	4	1916362	9.83 × 10^−10^	9.01	*Vigun04g024900.1*	Protein tyrosin kinase
	Vu04_2001620	4	2001620	5.89 × 10^−9^	8.23	*Vigun04g025900.1*	Chlorophyllase
	Vu04_2535911	4	2535911	1.26 × 10^−8^	7.9	*Vigun04g031500.1*	Auxin efflux carrier family
	Vu04_5101729	4	5101729	1.65 × 10^−8^	7.78	*Vigun04g054000.1*	Myb-like DNA-binding protein
	Vu04_41757989	4	41757989	5.05 × 10^−9^	8.3	*Vigun04g193600.1*	Serine/threonine-protein kinase
	Vu04_41787263	4	41787263	1.13 × 10^−8^	7.95	*Vigun04g193700.1*	NAD dependent epimerase/dehytrase
	Vu04_41800162	4	41800162	1.90 × 10^−9^	8.72	*Vigun04g194000.1*	Universal stress protein family
	Vu04_41850683	4	41850683	2.13 × 10^−8^	7.67	*Vigun04g194100.1*	3-hydroxyisobutyrate dehydrogenase-related
	Vu05_2631192	5	2631192	6.54 × 10^−9^	8.18	*Vigun05g032800.1*	Transferase family protein
	Vu06_10043938	6	10043938	3.87 × 10^−10^	9.41	*Vigun06g021500.1*	Coiled-coil regions of plant-specific actin-binding protein
	Vu06_30560091	6	30560091	2.52 × 10^−8^	7.6	*Vigun06g186400.1*	Transcriptional repressor
	Vu11_1322049	11	1322049	1.02 × 10^−8^	7.99	*Vigun11g010800.1*	Leucine-rich repeat
	Vu11_23659412	11	23659412	8.96 × 10^−10^	9.05	*Vigun11g080000.1*	Serine/threonine-protein kinase

**Table 2:** List of candidate genes having functional annotations that are relevant to plant abiotic stress. Protein homologs from each translated transcript was search in the cowpea (Vun), soybean (Gma), common bean (Pvu), and Medicago truncatula (Mtr) genomes. The number of protein homologs with similarity > 90% to that one from cowpea is reported.

Traits	Gene_ID	Functional_Annotations	Vun	Gma	Pvu	Mtr
Leaf SPAD chlorophyll under salt stress	*Vigun01g086000.1*	GATA transcription factor	1	4	2	1
	*Vigun02g128700.1*	Inorganic phosphatase	1	2	1	1
	*Vigun02g128900.1*	NAD dependent epimerase/dehydratase	5	12	5	3
	*Vigun02g129000.1*	NAD dependent epimerase/dehydratase	6	8	5	2
	*Vigun02g129100.1*	NAD dependent epimerase/dehydratase	2	9	4	3
	*Vigun02g129200.1*	NAD dependent epimerase/dehydratase	2	5	2	1
	*Vigun02g129300.1*	NAD dependent epimerase/dehydratase	4	5	2	1
	*Vigun02g129400.1*	NAD dependent epimerase/dehydratase	3	5	2	1
	*Vigun02g129500.1*	NAD dependent epimerase/dehydratase	3	5	2	0
Relative tolerance index for chlorophyll	*Vigun01g086000.1*	GATA transcription factor	1	2	2	1
	*Vigun02g129000.1*	NAD dependent epimerase/dehydratase	3	12	5	3
	*Vigun02g129100.1*	NAD dependent epimerase/dehydratase	2	9	4	3
	*Vigun02g129200.1*	NAD dependent epimerase/dehydratase	3	5	3	1
	*Vigun02g129300.1*	NAD dependent epimerase/dehydratase	3	5	2	1
	*Vigun02g129400.1*	NAD dependent epimerase/dehydratase	4	5	2	1
	*Vigun03g118000.1*	Terpene synthase	1	1	1	1
	*Vigun04g193500.1*	Phospholipid-transporting ATPase-related	0	2	1	0
	*Vigun10g093500.1*	Xanthoxin dehydrogenase/Abscisic acid biosynthesis	1	3	2	1
	*Vigun10g104200.1*	Cytochrome P450	9	8	3	4
	*Vigun10g104300.1*	Cytochrome P450	9	9	2	4
	*Vigun10g104400.1*	Cytochrome P450	9	7	3	3
Leaf injury score	*Vigun01g085400.1*	No apical meristem (NAM) protein	1	4	2	1
	*Vigun01g086000.1*	GATA zinc finger	0	0	1	0
	*Vigun02g129000.1*	NAD dependent epimerase/dehydratase	1	4	4	2
	*Vigun02g129200.1*	NAD dependent epimarase/dehydratase	3	5	3	1
	*Vigun03g135800.1*	Vacuolar iron transporter	3	7	3	4
	*Vigun03g136300.1*	EamA-like transporter family/phosphate antiporter	4	5	4	3
	*Vigun03g144700.1*	Potassium channel/Ion Channel	0	2	1	0
	*Vigun03g149400.1*	gibberellin 2-oxidase	1	5	2	2
	*Vigun04g025900.1*	chlorophyllase	0	3	1	0
	*Vigun04g054000.1*	Myb-like DNA-binding protein	1	2	1	0
	*Vigun04g193700.1*	NAD dependent epimerase/dehytrase	0	1	1	1

## Data Availability

Data are contained within the article.

## Supplementary Materials

The following supporting information can be downloaded at: https://www.mdpi.com/article/10.3390/ijms242015281/s1.
